# Deformable part models for object detection in medical images

**DOI:** 10.1186/1475-925X-13-S1-S1

**Published:** 2014-02-28

**Authors:** Klaus Toennies, Marko Rak, Karin Engel

**Affiliations:** 1 Computer Vision Group, Department of Simulation and Graphics, Otto-von-Guericke University Magdeburg, P.O. Box 4120, 39016 Magdeburg, Germany; 2 Leibniz Institute for Neurobiology (LIN), Center for Learning and Memory Research Brenneckestraße 6, 39118 Magdeburg, Germany

## Abstract

**Background:**

Object detection in 3-D medical images is often necessary for constraining a segmentation or registration task. It may be a task in its own right as well, when instances of a structure, e.g. the lymph nodes, are searched. Problems from occlusion, illumination and projection do not arise, making the problem simpler than object detection in photographies. However, objects of interest are often not well contrasted against the background. Influence from noise and other artifacts is much stronger and shape and appearance may vary substantially within a class.

**Methods:**

Deformable models capture the characteristic shape of an anatomic object and use constrained deformation for hypothesing object boundaries in image regions of low or non-existing contrast. Learning these constraints requires a large sample data base. We show that training may be replaced by readily available user knowledge defining a prototypical deformable part model. If structures have a strong part-relationship, or if they may be found based on spatially related guiding structures, or if the deformation is rather restricted, the supporting data information suffices for solving the detection task. We use a finite element model to represent anatomic variation by elastic deformation. Complex shape variation may be represented by a hierarchical model with simpler part variation. The hierarchy may be represented explicitly as a hierarchy of sub-shapes, or implicitly by a single integrated model. Data support and model deformation of the complete model can be represented by an energy term, serving as quality-of-fit function for object detection.

**Results:**

The model was applied to detection and segmentation tasks in various medical applications in 2- and 3-D scenes. It has been shown that model fitting and object detection can be carried out efficiently by a combination of a local and global search strategy using models that are parameterized for the different tasks.

**Conclusions:**

A part-based elastic model represents complex within-class object variation without training. The hierarchy of parts may specify relationship to neighboring anatomical objects in object detection or a part-decomposition of a complex anatomic structure. The intuitive way to incorporate domain knowledge has a high potential to serve as easily adaptable method to a wide range of different detection tasks in medical image analysis.

## Background

### Motivation

Although medical images provide insight into patient-specific human anatomy otherwise not accessible, their interpretation still requires extensive expert knowledge to fill the semantic gap between image data and depicted anatomy and/or function. Image characteristics of the object-of-interest may be known but are often not unique. Furthermore, noise, imaging or reconstruction artifacts alter the depicted content to a much greater extent than for pictures such as photos. Anatomical objects are not always contrasted well against each other and neighboring structures have similar appearance. However, most of the data is 3-D. Change of shape and appearance due to projection has not to be dealt with.

Still, detection and segmentation is possible if a suitable model completes missing information and corrects measurement errors in the data. The premier effect of such deficient information would be an incorrect delineation of the object's boundary. Hence, shape is the most important supplementary model information. Context information about adjacent structures, as well as information about object and background appearance may be added for discriminating among similar objects.

Developing a different method for each new detection or segmentation task is inefficient. Thus, a major challenge is to find a parameterizable representation that can be adapted efficiently for finding different objects.

Object shape can be represented by sampling points on its boundary. If such model is fitted to the data, boundary points of a model instance are registered with likely boundary locations in the image (e.g., high gradient strength locations). If artifacts or low contrast cause some model points not to have counter parts in the image, the model instance predicts the local course of the boundary there. Visible shape parts have to be sufficiently characteristic so that this prediction does not result in inacceptable errors.

Several problems have to be dealt with:

- The model has to include within-class variation of the structure of interest, while inhibiting influences from between-class variation.

- A weight for combining data and model needs to be set appropriately.

- The search for object instances in the data has to deal with many local minima of the corresponding optimization criterion.

Fusing shape model and image data requires a quality-of-fit (QoF) function where supporting information from the data are represented by a term $E_{external}$, which is regularized by a term $E_{internal}$, representing, in this case, deviation from the prototypical shape model:

(1) $$E=E_{internal}+\lambda \cdot E_{external}$$

This expression is found in many formulations of segmentation and detection tasks. It may be interpreted as an energy balance where external influences from a potential field generated by the data are counteracted by internal forces that represent domain knowledge such as the shape of an object or just the smoothness of its boundary. The parameter *λ *weighs the reliability of these two kinds of information. The equation can also be interpreted as logarithm of the conditional probability given by a normal-distributed likelihood function based on $E_{external}$ and a normal-distributed a priori probability depending on $E_{internal}$. Here, *λ *represents the ratio of variances of the two distributions.

Equation (1) may be optimized by gradient descent. It requires that the model instance is initialized sufficiently close to the optimum searched for. For global optimization, stochastic search techniques, e.g., the method by [1] may be used, which draws randomly distributed initializations and selects the best fitting candidates based on (1).

With this paper, we present an elastically deformable model as representation for shape and appearance. We argue that this model may be used efficiently for object detection as it does represent shape variation and expected appearance for an object class without requiring training (although training may improve performance). The main reason for this lies in the fact that model information is represented by integrating simple deformable shapes in a part-based model that, depending on the task, either represents object parts or guiding structures. We show that this model can be applied to solve different detection and segmentation tasks in medical image analysis.

The remainder of the paper is structured as follows. We first present previous work on models in object detection for medical images. We then present our elastic model, its application to object detection and its extension to a part-based model. We conclude with a number of examples that show the different capabilities of applying the model to detection and segmentation tasks.

### Previous work

Extracting an object-of-interest from the image contradicts the usual assumption for segmentation that segment characteristics are part of the data information. Extracting, e.g., a liver from CT images may require separating it from gall bladder, stomach, and feeding vessel. Depending on imaging modality and protocol the appearance of these structures may be very similar and insufficient for separation.

Extraction may be simple for a human observer with necessary knowledge about shape, appearance and location of the organ. In a computer-guided solution, a detection task has to be solved first, which supports the subsequent boundary delineation task. The two tasks may be solved in parallel or sequentially. In the latter case, the detection result constrains the subsequent segmentation. In the former case, a model instance is expected to deform into the object that is searched for.

The information that is needed for detection and segmentation can be quite complex. Necessary domain knowledge may be introduced at several stages of the process and the "intelligence" of the solution lies in the construction of the process from its sub-tasks (e.g., the kidney detection and segmentation scheme of [2]). Alternatively, a model can be built that contains information of all aspects about the object necessary for identification in the data including possible dependencies between different types of information. Detection is then fitting a model to measured data (e.g., the application of a shape and appearance model for detecting the left ventricle of the heart in MRI [3]). We prefer the latter approach as it separates model description from the search for model instances and may be adapted easier to a new detection task.

If detection precedes delineation, it localizes the object-of-interest. In this case, the result locally constrains boundary delineation if the object is not sufficiently contrasted against adjacent structures. Localization may be interactive (e.g., seed points for region growing [4], closed active contours [5], or starting points for live wire delineation [6]). In this case, the detection result often includes little information with respect to object boundary, since interaction costs are usually kept to a minimum. Hence, interactive localization is most suitable if the boundary itself is well defined by the data and interaction is used to find the object among different structures with similar appearance. Small artifacts such as noise can be accommodated by requiring smooth and closed boundaries. If substantial parts of the boundary cannot be derived from data information, they need to be added by interactive delineation of boundary parts.

Localization by matched filter methods such as vesselness filters [7], blobness [8], template matching [9] and Hough transform [10] does not require interaction, except possibly for specification of a relevancy threshold for a successful detection. These methods are capable of finding multiple instances of an object. The filters predict the (average) object shape and appearance which may then be used as prior for data-driven segmentation, e.g., by graph cuts [11-13] or level sets [14,15]. The methods operate with few parameters which usually can be found easily. Of course, it restricts shape representation either to variable, but simple shapes (e.g., the vesselness filter) for which intra-class variation can be described by few parameters, or to a representation by a single average shape (e.g., the generalized Hough transform).

Representing acceptable shape variation of complex object shape is more difficult. Unconstrained shape variation will cause the model to fit almost everywhere in the image while an overly constrained shape will not fit well to the data. Active Shape Models (ASM) and its many variants have found widespread use since their introduction in [16]. Shape is represented by coordinates of a set of labeled points. If appearance variation shall be represented as well, it is given by intensity or texture variation at these points. Variation is trained from correspondingly labeled test data. Influence from rotation, translation and sometimes scaling has to be removed by Procrustes analysis [17]. Variation is assumed to be normal-distributed and highly correlated. Hence, principal component analysis is used for de-correlation and information is reduced to a few eigenmodes (modes of variation). The model enables a potentially arbitrarily exact fitting of the missing boundary, as long as the visible part of the boundary sufficiently constrains the variation of the missing part.

Training of such a point distribution model (PDM) can serve two purposes. It estimates shape (and appearance) variation of the class of objects to be delineated and it restricts shape variation. The success of PDMs for model-guided segmentation has been shown in numerous publications (e.g., [5,18,19]). Often only few training samples - considering the degrees of freedom of the untrained model - are needed for segmentation. The reason is probably twofold. Firstly, within-class shape variation has much fewer degrees of freedom than the model so that the true co-variance can be estimated from few samples. Secondly, boundary delineation does not require exact estimates of intra-class variation anyway as long as incorrectly estimated shape variation is corrected from boundary information in the data.

Besides PDM, the Hough transform may be extended into a probabilistic shape model as well [20]. However, compared to a PDM, its versatility is restricted since appearance information is not part of the model and it is unclear how the trained information may be used for subsequent boundary delineation.

Since a shape model has not to be very accurate for boundary delineation, prototypical variation has also been used for shape-supported boundary delineation. If detection is not necessary (e.g. when tracking a heart shape in a 3d+time sequence), the necessary shape information are properties such as closedness, smoothness and similarity of the boundary between time frames. This has been used, e.g., in tracking the heart beat from CT using an active surface based on a finite element model (FEM) to restrict boundary variation between time steps [21]. This concept has been extended to an elastically deforming shape model (e.g., the mass-spring model [22] or an FEM [23]). Such model restricts organ variation to elastic deformation of a prototypical shape. This is particularly simple for a homogeneous, linear-elastic FEM since only few parameters regulate its stiffness. Defining such a prototype solves part of the optimization problem in detection as well, since - similar to active contour models - rather basic image information can be embedded by forces acting on a model instance, locally attracting it to the object-of-interest.

However, an elastic model provides just an approximation of intra-class shape variation. To some extent, either the delineation goal or the detection goal has to be sacrificed if the shape of the object itself or its variability is complex. If stiffness overestimates true shape variation, the model instance may adapt to the object boundary given sufficiently reliable image information, but shape deformation may not be used for object detection. If the shape variation is overconstrained it may be used for object detection but will result in a poor segmentation of the object.

Instead of training complex shape variation, some of this variation can be efficiently represented using part-based models. A shape is decomposed into simpler parts. Variation is then that of the parts and that of part-relationships. In other applications, part-based models have been successfully used for articulated object detection (e.g., detection of faces and people [24], or pedestrian recognition [25]). Although part-relationships may be learnt [26], an advantage is that the qualitative knowledge of a decomposition of an object into parts is often readily available (e.g. the decomposition of the spine into a sequence of vertebrae separated by disks). We successfully applied a combination of deformable models with a part-based representation for several object detection and boundary delineation tasks in medical image analysis.

With this paper we describe how to use our part-based deformable model for object detection and segmentation and illustrate the applicability to different tasks in medical image analysis.

## Methods

As stated above, variation represented by deformable models often does not have to be exact. However, if it is to be specified by the user instead of being trained from samples, parameterization of the model should be intuitive. Representing the object as an elastically deformable material meets this condition since most users have an understanding of how elasticity influences shape variability. Elastic models have been used in medical image applications for quite some time (e.g., [27] for elastic registration), although not often to restrict shape variation for detection tasks. We use this concept to replace trained shape variation. It should be noted that we do not want to simulate deformation behavior of an object (e.g., the deformation of the heart ventricles over time). Elastic deformation is solely used to replace distribution estimates to restrict shape variation among objects of the same class. Since describing complex shape variation by few elasticity parameters may be too simple, we use deformable objects as components of a part-based model. Relationships between parts are modeled by an elastic superstructure.

Object detection consists of a local and a global part. Localized delineation lets a model instance be attracted and deformed by image features. The global search is realized by repeatedly generating random initializations of model instances and selecting the best fitting candidates. Fitting is defined according to equation (1) by a data term that is regularized by shape deformation.

### Finite Element Models (FEM)

We use FEMs to represent the deformable shape. A finite element model represents linear-elastic deformation of a 2-D or 3-D object based on a discretization of the object domain into finite elements *e*. Elements are generated from node locations that sample the object domain. The number of nodes depends on the accuracy with which an object is described. For a given object, the FEM is generated from a 2-D or 3-D representation of the object's shape, either based on a priori knowledge, e.g., about the shape of a vertebra, or from an example segmentation. If segmented data is used, it should reflect a representative object example. It may also be helpful to smooth the segmentation in order to remove irrelevant detail from the model instance before generating the FEM. The FEM may be generated by triangulating the segment, e.g., by computing a Delaunay triangulation.

For a single element, external forces $f^{\left(e\right)}$ at element nodes cause node displacement $u^{\left(e\right)}$. Their relation is governed by a stiffness matrix $K^{\left(e\right)}$

(2) $$K^{\left(e\right)}u^{\left(e\right)}=f^{\left(e\right)}.$$

The displacement depends on applied forces, the elasticity and geometry of the element, as well as on the interpolation functions used to compute continuous material deformation within the element. When linear interpolation is sufficient - which is true for our applications - element shape and elasticity parameters are the only influences that define the deformation behavior.

The different elements *e *of the FEM are assembled according to the decomposition of the object domain (for details see [28]). It results in an aggregated stiffness matrix **K **which gives the following relation between forces **f **and displacement **u **via

(3) Ku=f.

**Figure 1:**
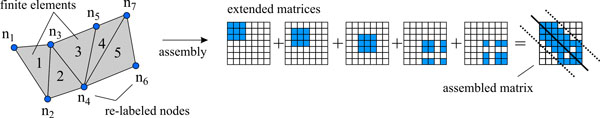
**Assemblage of an FEM from elements**. Elements are assembled by first re-labeling nodes, then extending the stiffness matrices accordingly (non-zero entries are indicated in blue) and then adding the matrices.

Element-wise assemblage happens in two steps (see Figure 1). First, nodes of all elements are re-labeled so that nodes of two different elements have the same label only if this node is common to the two elements. Then, expanded matrices $K_{exp}^{\left(e\right)}$ and force vectors $f_{exp}^{\left(e\right)}$ are created for each element *e *having a size that depends on the total number of nodes of the FEM. Locations in $K_{exp}^{\left(e\right)}$ and $f_{exp}^{\left(e\right)}$ that represent labels not occurring in *e *are filled with 0's. Assemblage is then simply summing expanded matrices and vectors.

**Figure 2:**
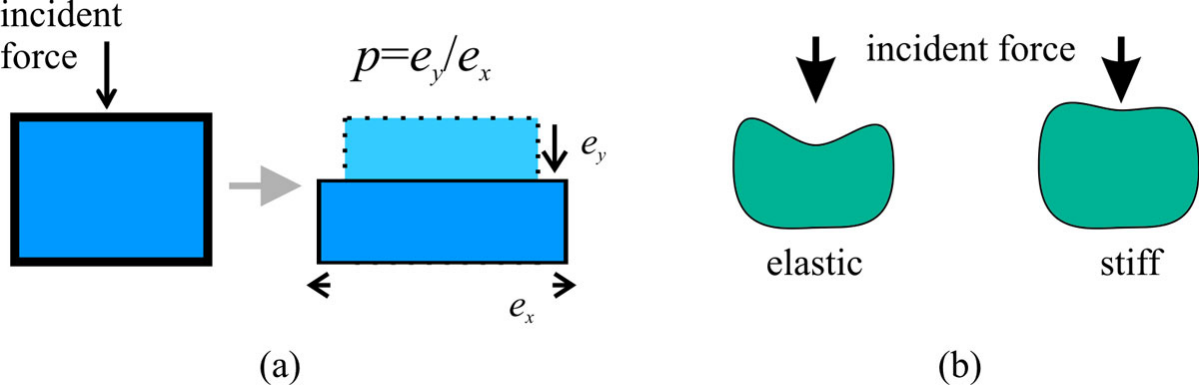
**Factors that influence the elasticity of an FEM**. (a) Poisson ratio p represents how displacement due to an incident force is transferred orthogonal to the direction of incidence. In (a) the Poisson ratio is negative, since a contraction in force direction causes an extension orthogonal to it. (b) Elasticity represents the amount of deformation caused by an incident force.

The FEM approximates the mean shape and possible physics-based deformation of the object-of-interest. Shape variation among different exemplars from the object class is given by the FEM decomposition and few material parameters. Assuming that the model consists of isotropic, linear elastic material, the elasticity of the elements can be described by Poisson's ratio and the elastic modulus [29]. The Poisson's ratio *p *is the ratio between deformation in the direction of an incident force and deformation orthogonal to it (see Figure 2a). For most materials, 0 ≥ *p *> -1, since compression in one direction causes expansion in the orthogonal direction. For object detection, it may be useful to define a positive Poisson ratio. This would cause contraction in all directions if forces in some direction cause compression. Scale invariance can be implemented (to a small extent) using this parameterization.

The elastic modulus, given the isotropic material assumptions above, may be described by Young's modulus. It tells how an isotropic material resists deformation due to opposing forces (see Figure 2b). Changing Young's modulus controls the amount of deformation due to external forces, e.g., image-derived forces.

Given element geometry, interpolation functions, Poisson's ratio and Young's modulus, the stiffness matrix can be computed for each element and assembled into global stiffness matrix **K**. Hence, the FEM, created from an exemplary segmentation or from a priori knowledge, represents a deformable shape by very few parameters that are intuitive in a sense that a user does not have to understand the underlying numerical concepts of the FEM method.

### Dynamic optimization and object detection

Using the FEM for object detection requires localizing and deforming the model based on image-derived forces. The extension of the FEM to a dynamic model has the practical advantage that parts of the localization tasks can be solved as a time-dependent attraction of a model instance to local image features. It requires extension of the governing equation by a mass matrix **M**, which represents resistance to forces based on the current acceleration **ü**(*t*) at time *t*, and by a damping matrix **D **that represents transfer of kinetic energy dependent on current speed $\dot{u}\left(t\right)$:

(4) $$M\ddot{u}\left(t\right)+D\dot{u}\left(t\right)+Ku\left(t\right)=f\left(t\right).$$

Specifying masses in **M **is necessary if a moving model instance should resist force changes from the image in order to let it move over spurious image details from noise or artifacts. If this is not necessary, mass may be omitted, leading to a damped gradient descent.

For practical reasons, we use Raleigh damping, which defines **D **as linear combination of **M **and **K**:

(5) D=αM+βK.

If the system is mass-free, we may set **D **= **I **+ *β***K**, where **I **is the identity matrix.

External forces acting on FEM nodes may be defined separately and differently for every node. It is, however, practical to define node groups and let each node group be attracted by the same kind of image forces. Examples are the following:

- Boundary nodes are attracted by boundary features in the image

- Inner nodes are attracted by image features according to the specific appearance of the object to be detected

Different kinds of boundary or appearance nodes can be defined if the expected edge strength or appearance varies in an object-specific fashion.

External forces can be computed based on the current location and displacement of nodes with respect to image features in the model's vicinity. This may become necessary if the data is unreliable and current node locations and displacement are used to select image regions evaluated for force computation. An example will be demonstrated in Application 2 described below.

**Figure 3:**
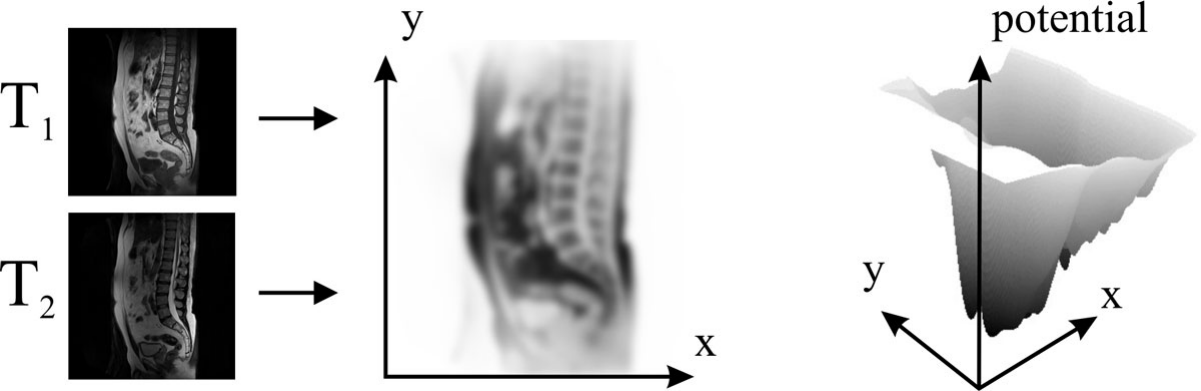
**Example of a potential field**. The field is generated from a weighted combination of a T1- and a T2-weighted image for attracting nodes to vertebrae. It is clearly visible that the minima for the vertebrae are just local minima, since several internal organs produce a much more pronounced minimum. Detection will therefore require additional information from shape and configuration of vertebrae.

If data quality permits, external forces can be pre-computed as gradient of a potential field derived from the image. Potential fields may be defined separately for each node group (see Figure 3). Nodes of the FEM are pulled to a minimum in the potential. In order to attract an FEM instance over some distance, the potential field is convolved with an influence function, i.e., a low pass filter that is decreasing with distance from the kernel center. Local features for boundary nodes are edges or edge features such as gradient length. Local features for appearance nodes depend on expected intensity or texture. The influence function is a Gaussian of which the variance determines the influence radius.

It should be noted that, while boundary nodes relate visible object boundary in the image directly to model nodes, appearance nodes typically do not. Since expected appearance (e.g., brightness) may or may not be constant over the object it may not help to move and deform an FEM instance into its proper place. Appearance nodes are useful nonetheless:

- A single or several appearance nodes may help to determine whether the boundary nodes - which are only sampling the boundary - sit on the correct object boundary.

- Appearance nodes that sample the appearance inside the object and in the background in the object's vicinity may serve as a complex boundary model in cases where the boundary is difficult to detect in the data using a local operation such as edge detection.

Since local features are not unique (otherwise, detection would be trivial), the influence radius, i.e., kernel width should be as large as possible in order to attract FEM instances from far away, but small enough to avoid overlapping influences, i.e., blurring from different local features.

Given particular potential fields, an FEM instance can be placed anywhere in the image and deforms under the field-derived forces. The potential field is constant over time and may be pre-computed. Forces **f **change with the displacement in the potential field and, therefore, with time. An instance has found its final destination when internal forces from deformation and external forces from the image balance each other. Computing adaptation of the deforming FEM instance to the image requires computation of the dynamic system described by equation (4).

Computation of this system of dependent differential equations can be done in several ways (see, e.g., [28]). We prefer a computation via decorrelation into independent modes. It does not only produce a stable solution to the problem but also allows selecting relevant deformation modes (similar to the selection of variation modes in ASM). This has been used for object classification [30], mapping between similar objects [31], and to provide a base for training an ASM, if training is desired and possible [32].

For optimization, a generalized eigenproblem is solved for mass matrix **M **and stiffness matrix **K**

(6) $$KE=ME\Lambda \;\text{with }E^{\text{T}}KE=\Lambda \;\text{and}\;E^{\text{T}}ME=I,$$

where **Λ **is the diagonal matrix of eigenvalues, **E **is the column matrix of eigenvectors and **I **is the identity matrix. Mapping **K **and **M **onto **E **and assuming Raleigh damping according to equation (5) results in a new system

(7) $$\begin{array}{c} M^{\prime }\ddot{c}\left(t\right)+D^{\prime }\dot{c}\left(t\right)+K^{\prime }c\left(t\right)=E^{\text{T}}f\left(t\right)\\ \text{with }M^{\prime }=E^{\text{T}}ME=I,D^{\prime }=E^{\text{T}}DE=\text{diag }\left(d_{1},...,d_{n}\right),K^{\prime }=E^{\text{T}}KE=\Lambda \\ \text{and }Ec\left(t\right)=u\left(t\right),\;E\dot{c}\left(t\right)=\dot{u}\left(t\right),\;E\ddot{c}\left(t\right)=\ddot{u}\left(t\right). \end{array}$$

The new diagonalized system contains simple differential equations of the type

(8) $${\ddot{c}}_{i}\left(t\right)+d_{i}^{\prime }{ċ}_{i}\left(t\right)+\lambda_{i}c_{i}\left(t\right)={e_{i}}^{\text{T}}•f_{i}\left(t\right),$$

where **e**_i _is the *i*-th eigenvector of **E **and *λ*_i _is the *i*-th eigenvalue. An analytical solution for such equations can be computed stably and fast. The approach is applicable for a mass-free system as well.

Eigenvectors and eigenvalues carry semantics similar to ASM. An eigenvector is called a *mode of vibration *and represents a generalized symmetry axis of shape deformation. The overall deformation is a weighted sum of the generalized deformations due to the transformed forces **E**^T^**f**(*t*). It is possible to reduce the number of modes in order to remove small information details and reduce computational costs. Eigenvalues characterize the amount of force necessary to cause displacement. Assuming ascending order of eigenvalues, the first *n*! eigenvalues of an *n*-dimensional FEM will be zero and represent rigid transformation (rotation and translation). The following, intermediate eigenvalues are the most relevant modes representing shape deformation.

**Figure 4:**
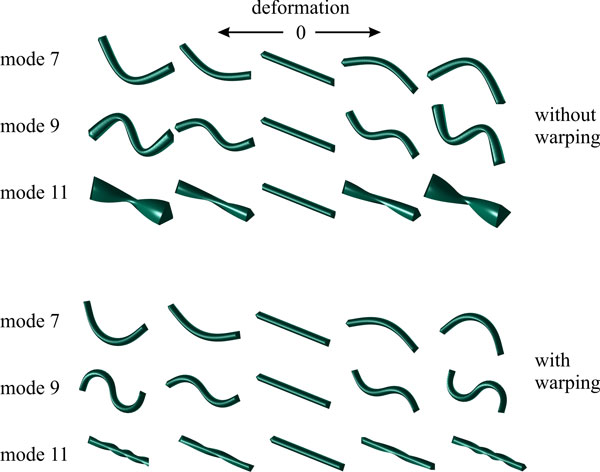
**Modes of vibration of a 3-D stick model**. The first *n*! modes of an n-d model (in this case the first 6 modes of the 3-D model) represent rotation and translation and are not shown. Only odd modes are shown since, in this case, deformation of mode 2k is the same than mode 2*k*-1, except that it deforms orthogonal to mode 2*k*-1. If warping is omitted (upper row), local rotation is resulting in unwanted deformation.

Rigid transformation is still part of the model, which is different to ASM where the trained model is normalized with respect to rotation and translation and where this information has to be incorporated into the fitting process by means of an additional registration step. This causes problems, however, since deformation constrained by the stiffness matrix **K **is defined as strain caused by a directed force on an infinitesimal line at this point. Since this line is defined in the given coordinate system, rotation of the FEM instance would make the stiffness matrix **K **dependent on the current rotation, hence dependent on time *t*. This is unwanted since it would require re-computation of **K **and of the vibration modes at every new iteration step. Neglecting it would cause some part of the rotation to be interpreted as deformation possibly leading to serious distortions (see Figure 4) [33]. This is only acceptable if we assume that local fitting of the model instance comprises only little rotation. Still, this would result in a loss of the benefit of including rotation into the optimization. Hence, we use warping techniques, presented, e.g., by [33,34] to circumvent the problem.

The basic idea of warping is to include the current rotation into the representation. Therefore, forces are applied to the un-rotated model and the result is rotated back. If warping is applied on the node level, the current rotation **R***_i _*is computed for all nodes *i*. They are concatenated into an orthogonal matrix **R **of which its inverse is applied to the current displacement **u**(*t*) as well as to its derivatives. Deformation is then applied to these vectors and the result is rotated back (the impact of this correction can be seen in Figure 4). It can be shown that this can be applied in the spectral basis as well [33]. This essentially means that the current vibration modes are "rotated" versions of the original modes.

The current rotation can be computed by several methods. A fast way is to search for the rotation **R***_i _*at node coordinates **x***_i _*that optimally registers difference vectors **d***_ij _*= **x***_i _*- **x***_j _*between **x***_i _*and adjacent nodes **x***_j _*relative to difference vectors **d**_*ij*0 _at *t *= 0. The resulting optimization problem

(9) $$R_{i}=\underset{R\left(t\right)}{\text{arg min}}\sum_{j=1}^{M}∥d_{ij}\left(t\right)-R\left(t\right)d_{ij0}∥$$

for each node can be computed fast using a method proposed by [35].

Dynamic optimization of an FEM instance as described above produces a locally optimal fit. For global optimization we use a stochastic initialization technique similar to the method that was suggested by [1]. FEM instances are initialized at different locations in the image and serve as detection candidates for the object-of-interest. After performing local optimization as described above the best candidates spawn new instances in their vicinity. The process ends when no further improvement can be reached.

Rating candidates requires definition of a QoF function. It is defined according to equation (1). Data quality $E_{external}$ measures the value of the potential field at final node locations and the regularization term $E_{internal}$ measures the deviation of the model from its initial shape based on the weights of the vibration modes.

Computational complexity of the process is *O*(*N*) where *N *is the number of nodes of the model instance. However, computation times depend on the convergence of the various iterative optimization schemes, namely the iterative deformation, the iterative computation of the singular value decomposition in [35] for computing the warping, and on the number of iterations in the stochastic search.

### Hierarchical part-based model

The few parameters of the deformable model described in the previous section are sufficient for object detection as long as the object in question has a rather characteristic mean shape and appearance. If this is not the case, training of shape variation such as in ASMs would help. However, to reduce training effort (ideally up to a point where all parameters are user-specified based on domain knowledge), information has to be included in a qualitative rather than a quantitative way. Hence, we adopted the principle of part-based models for augmenting the descriptive power of our model.

Representation by a part-based model serves two different purposes:

- The parts may be sub-objects of the object-of-interest (such as the vertebrae of a spine model).

- The parts may relate the object-of-interest to surrounding objects that help to localize the object.

Additionally to the elastic deformation of the parts, division into parts, their relationship to each other, and potential mutual deformation are relevant for model specification.

A part-based model can be realized conveniently within a hierarchical FEM (HFEM) framework [36-38]. The shape is decomposed into parts based on user's specification. Each part is represented by an FEM. This constitutes the morphological layer of the complex object. Parts may have different material properties and different potential fields to accommodate their different semantics. Spatial relationships between parts are represented by a second-level FEM which constitutes the structural layer. Forces on the structural layer FEM are exerted from deformation and displacement on the morphological layer of the HFEM. They, in turn, impose constraints on the shape of its structural layer.

The HFEM may be realized explicitly by generating a set of morphological and structural FEM or implicitly by generating a single, heterogeneous FEM from the morphological and the structural FEM.

**Figure 5:**
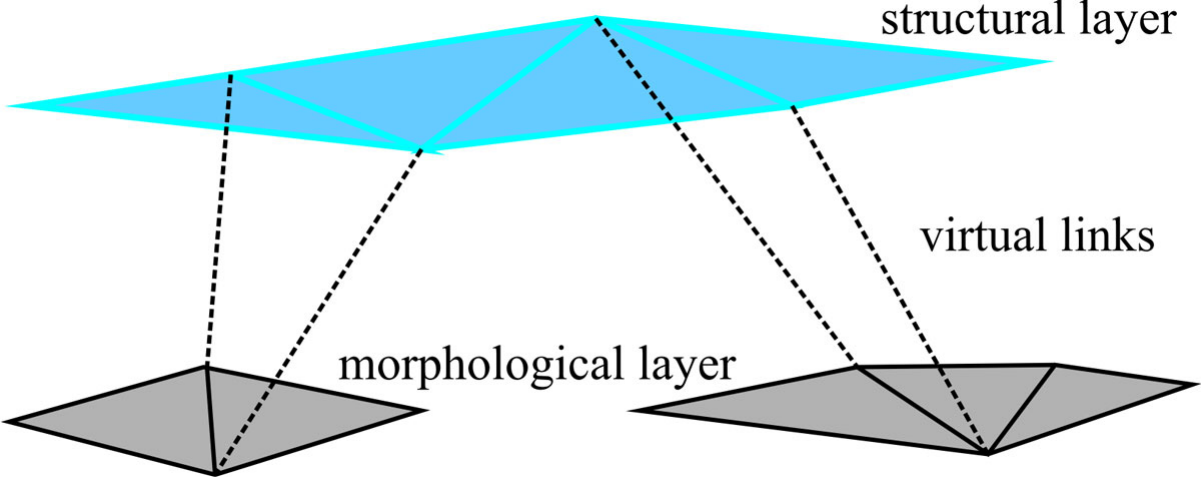
**Hierarchical FEM**. The structural layer is connected to the morphological sub-shapes via virtual links. Each displacement from one layer is transferred as force on the other layer.

**Figure 6:**
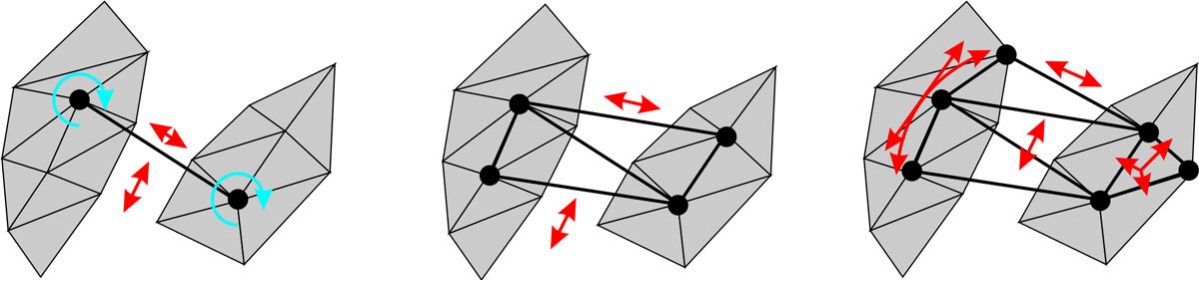
**Different kinds of connection between layers cause different kinds of restrictions**. (a) Single-connected nodes allow for independent rotation around the nodes. (b) Double-connected nodes can be used to enforce approximate orthogonality or parallelism (this example) between sub-shapes. (c) Connecting further nodes of the sub-shape with the structural layer enforces co-deformation, such as, e.g., curvedness of the sub-shape (left sub-shape in 6.c) or its size (right sub-shape in 6.c).

Explicit representation results in a set of independent morphological FEM that are coupled to the structural layer via virtual zero-length-springs that connect nodes of the two layers [36] (see Figure 5). Relationships between sub-shapes are specified by the kind of connection between the layers. For instance, the following relationships can be defined for a 2-D model using different numbers of nodes (see Figure 6):

- A single-connected shape may rotate independently but is restricted in distance to other sub-shapes via the structural layer.

- A sub-shape that is connected via two nodes to the structural layer is further restricted in its rotation. Properties such as approximate orthogonality or parallelism of parts can be ensured by this kind of connection.

- A sub-shape connected by more than two nodes shares some of its non-rigid deformations via the structural layer.

The influence of external forces between layers is then realized by a message passing algorithm [37]. Displacements due to deformation on the morphological layer act as external forces on the structural layer, while deformations on the structural layer cause forces on the morphological layer. Computation of the dynamic behavior, hence, requires solving the optimization problem presented in the previous section for each sub-shape on the morphological layer, passing the resulting forces to the structural layer, computing the resulting deformation and passing displacement back to the morphological layer.

In the hierarchical representation, each FEM is diagonalized separately resulting in its own vibration modes. It allows selecting vibration modes independently for each FEM. Hence, different requirements on precision, e.g., between structural and morphological layer, or for different substructures on the morphological layer can be accommodated.

When computing the quality of fit, equation (1) has to be computed first for energies *E_j _*of each subshape *j *on the morphological layer, resulting in a weighted sum of morphological fits

(10) $$E_{total}=\sum_{j=1}^{N}w_{j}E_{j}.$$

The weights *w_j _*may be used to account for a different importance of parts. If, for instance, some parts are just guiding structures to find the actual object-of-interest, their fit has not to be very precise.

Since the part relationship may be decisive for detecting the object, deformation and image support is then computed for the structural layer FEM as well. Deformation is measured in the same way as for the morphological layer. The external energy is now given by the state of the morphological sub-shapes. Hence, it is simply defined by the QoF from the sub-shapes of the layer below.

**Figure 7:**
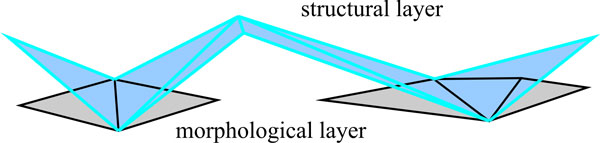
**Integration of morphological and structural sub-models in a single FEM**. Structural and morphological layer are connected and elements of the different FEM are assembled to a single FEM. Elasticity parameters of the morphological and structural FEM may be different.

Alternatively to representing the part model as a hierarchy of homogeneous FEM it may also be represented as single heterogeneous FEM [38] (see Figure 7). Heterogeneity refers to the fact that it will be a combination of the different part-FEMs, each of which possibly having different elasticity parameters.

Morphological and structural layer FEM are defined as above. However, instead of treating them as single FEM, which communicate through message passing, the FEM are assembled in the same way as elements were assembled to the FEM. Assemblage is guided by node connections between structural and morphological layer as before. Through the assemblage these nodes are treated as shared nodes between elements of the morphological and the structural layer.

Solving the part-based FEM optimization by assemblage in a single, heterogeneous FEM has different properties compared to the previous method:

- Computation of vibration modes applies to the complete part-based object and optimization does not require message passing between deformable part models.

- The quality-of-fit is computable in the same way than for a single FEM based on deformation and input from the data.

- Different potential fields and/or different material properties of the part-FEM replace the weights in equation 10.

- If warping is used on the node level this applies to the complete FEM. Hence, single-connected FEM do not allow independent rotation.

In general, the second solution is simpler regarding computation of dynamic deformation and stochastic optimization, but it also integrates parts tighter into the representation. Therefore, it depends on the application which of the two methods should be preferred. This will be discussed further in the next section where we will present different applications using the two approaches for different analysis goals in medical image analysis.

## Results and discussion

In the following, we present a number of applications to illustrate how to parameterize and use a part-based deformable model for various tasks in medical image analysis. We will summarize performance and results for each application. Since we use the application to demonstrate the use of the model, we refer to the original publications [38-40] for details.

### Indentifying Heschl's gyrus in flat maps of the human cortex

Experiments with functional magnetic resonance imaging (fMRI) attempt to localize and delineate particular brain regions, such as the human primary auditory cortex (pAC) and neighboring higher-order areas, in vivo. The pAC is known to be located on the first transverse temporal gyrus (i.e., Heschl's gyrus, HG). Since the region covered by the pAC is very small with respect to the spatial resolution of fMRI and the signal to noise ratio is rather poor, fMRI data of a population of subjects are combined to arrive at a representative functional map. The combination of individual data to a group map requires the mapping of corresponding regions across subjects. We solved this registration task by localizing macro-anatomical landmarks (i.e., the lateral "Sylvian" fissure and superior temporal sulcus) that delineate the superior temporal lobe and enclose the highly variable Heschl's gyrus. The detection is performed using a deformable model of HG that is fitted to the cortical surface (i.e., a surface mesh that represents the grey-white matter interface, gwI) [36,40]).

**Figure 8:**
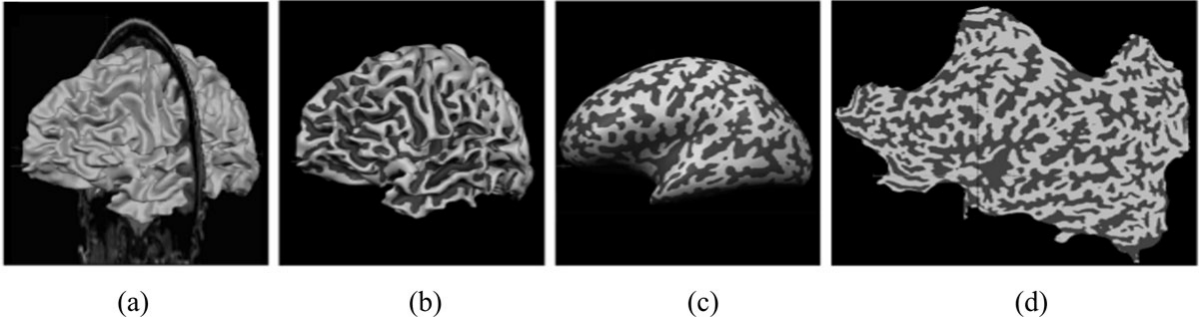
**Flatmap generation from anatomical MRI**. (a) reconstructed grey-white-matter interface (gwI) (b) overlayed curvature depicts gyri (light) and sulci (dark) (c) inflated gwI (d) flattened gwI.

Input data for the deformable object detection task are 2-dimensional flat maps of the gwI (details about cortex reconstruction and flattening can be found in [41]). The function value at each location in the flat map is the curvature of the folded cortex before flattening (see Figure 8). Hence, change from outward folds (gyri) to inward folds (sulci) is given by zero crossings in the flat map.

Identifying HG in a given flat map requires finding a specific U-shaped border at those zero crossings. This is difficult since the size, location, orientation and individual shape of HG vary substantially between subjects, while there are several gyri of similar shape in each cortical flat map. Experts identify the correct gyrus by means of its relation with two anatomical landmarks: the Sylvian fissure (SF) and sulcus temporalis superior (STS) are approximately parallel to each other and orthogonal to HG.

A part-based deformable model is well suited to represent anatomical descriptions such as U-shapedness and spatial relations between cortical gyri and sulci.

HG, STS and SF were modeled on the morphological layer of a two-level model and combined on a structural layer that models the structural configurations of the macroanatomical landmarks. Since the gyri and sulci were simple 2-D shapes, they were manually drawn based on example images. For HG, two different variants were created since some subjects may have a HG with a sulcus intermedius (SI).

Boundary nodes of the HG and the HG+SI models had access to smooth potential fields, whose minima represent zero crossing locations of the flat map. In practice, the filtering operations were approximated as follows. For a given surface mesh we computed a discrete, difference-of-Gaussian filtered version of the curvature mapping: We first applied an operator that separated convex regions (gyri) and concave regions (sulci), and then we used a discrete approximation of a heat diffusion kernel to smooth the resulting binary map. The low pass filter kernel widths of *σ*_1 _= 2 mm and *σ*_2 _= 2*σ*_1 _for the difference-of-Gaussian provided a good tradeoff between a large region of influence and blurring of adjacent zero crossings. During the model fit, we estimated for each model node the vector to the nearest salient vertex on the cortical surface mesh (with a local maximum filter response and within a given sampling distance) and used the weighted radial component of this vector as external force.

Internal nodes of the HG model responded to positive curvature (gyri) only, whereas the HG+SI model contained nodes responding to negative curvature (sulci) at the SI location as well. Again, the potential fields were in practice defined based on heat kernel smoothed versions of the binary curvature maps.

**Figure 9:**
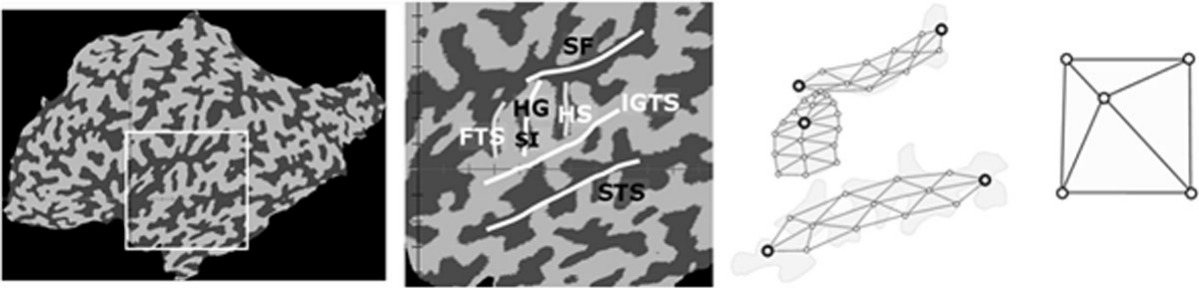
**Generation of the hierarchical model from an example image**. Models of Heschl's gyrus (in this case without a sulcus intermedius - SI), sylvian fissure (SF) and sulcus temporalis superior (STS) are connected via virtual links to the sparse structural model depicted on the right.

The shape and pose of HG, the exact relations between HG and the landmarks and also the shape of the landmarks SF and STS vary dramatically between subjects:

- Since SF and STS primarily served as limits for restricting the cortex region to be searched for HG, it was important to robustly and correctly localize these landmarks, while only roughly matching their main branches. The STS and SF models were constructed as simple line-shaped structures, undersampled with FE nodes that responded only to appearance, i.e. curvature information. This sparse sampling allowed bridging gaps in the curvature maps, while sufficient similarity of the structure to a line was still ensured. (example images showing variability of SF, STS)

- The model at the top-layer arranged HG (and HG+SI, resp.) as the central part of the AC folding pattern nearly orthogonal to the two surrounding parallel sulci. To set up a sparse top layer model, "link" nodes were identified for related sub-shapes and duplicated such that the resulting structural model consisted of these shared link nodes (see Figure 9). Four nodes represented the parallel arrangement of SF and STS in the 2-D flat maps, and the internal node was linked with the HG model to position it "above STS" and "below SF". In the structural prior model HG+SI an additional node was linked with a simple SI model in a "contained in HG"-relation.

- The FE nodes are then subject to boundary conditions, such that any nodal displacement in the morphological coordinate frames (e.g., due to image forces) enforced displacements in the global model coordinate frame, while the resulting nodal displacements of the top-layer model acted as across-level spring forces on the link nodes of the morphological shape models.

- The search for the independent optimum pose parameters (of the structural model as well as HG and HG+SI, resp.) was based on a quasi stochastic sampling of an a priori constrained search space. To define the constraints, we used the Talairach-transformed flat map coordinate system [40,42] and asked an expert to annotate an example and specify possible variations in the location, size and orientation of the anatomical structures. This information was encoded in terms of pose parameter distribution functions.

- Young's modulus was 2.0, Poisson ratio was 0.4 and material density was set to 1.0 for all models.

At each iteration of the deformable model search, a population of 100 model instances was initialized with random affine parameterizations and fitted to the data. Randomness was introduced into this evolutionary process by employing a rank selection of fitted instances. Reproductive success varied with the relative "fitness", i.e. quality, of instances. Poor fitting results were deleted, and Gaussian noise was added to the pose parameters of the best fitting instances to simulate the "mutation" in the reproduction step and initialize new deformable model instances. This evolution process ends if no improvement in overall fitting quality was observed (typically after 3 steps).

Success of the deformable model search for HG was measured by determining the percentage of cases were the correct gyrus was identified by one or more model instances among the 2% of best rated candidate solutions. The method was applied to flat maps from 80 subjects. A detailed discussion of results can be taken from [36,40]. In summary, we could show that

- Using just the HG model without guiding structures and constraints on possible poses resulted in a 5% success. By constraining the search space, the correct gyrus was identified in about 50% of all cases.

- Using the HG model together with SF and STS resulted in 80% success which can be improved to more than 90% if model parameters have been trained.

- This part-based model could be directly used (1) to compute precise segmentations of HG with less than 3 mm error compared with manual segmentations of HG and (2) to classify the given cortical surfaces correctly with respect to the presence of a sulcus intermedius.

- Using a single-layer model that comprises HG, STS and SF instead of the multiple layer model led to a significant decrease in result quality which did not improve by training. This means that the relevant anatomical information was better captured by the structural decomposition and deformation parameters.

- The method is very robust to changes in the parameterization.

This example shows that domain knowledge, such as the structural arrangement of landmark structures that predict the location of a highly variable object of interest within data that carry poor semantics, can be directly formulated in the form of efficient constraints of a hierarchical, deformable model. This is very interesting in that one could expect that such a model provides a better symbolic representation of the "true" object anatomy and anatomical variability than a model that is learnt from annotated training data. Moreover, the expert knowledge can be more efficiently improved by training (e.g., of correct poses) and expressed with less effort (e.g., by annotating a single example and "painting" connecting relations such as "parallel to" and "contained in" between the different structures, by accepting good solutions, applying interactive forces during the model fit or by correcting the deformed shape of poorly fitted model instances).

### Segmenting the Substantia Nigra in transcranial sonography

Transcranial sonography (TCS) produces ultrasound images of the brain that are acquired by imaging through the temporal bone window. The mesencephalic brain stem, or midbrain, containing the substantia nigra (SN) is visible in TCS images, although image quality compared to regular ultrasound images is poor.

**Figure 10:**
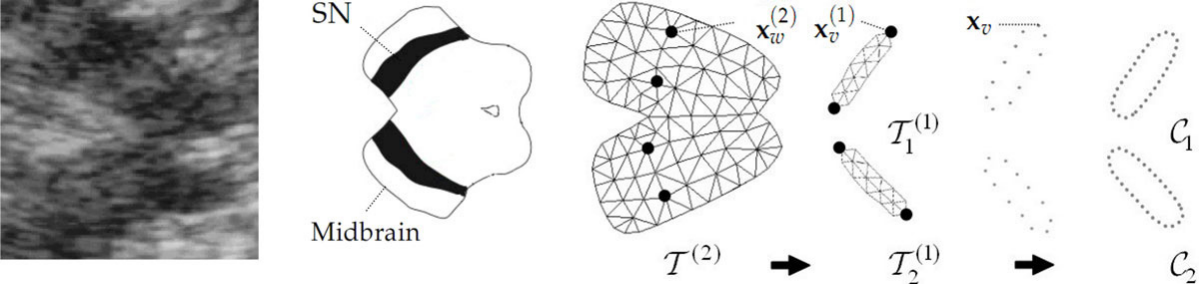
**TCS of the midbrain (left) and derived model (right)**. The model T(2) on the upper layer represents the midbrain morphology which has been attached to substantia nigra (SN) models T1(1) and T2(2). After fitting model instances to the data, the boundary nodes of the fitted Ti(2) model instances are selected and then subsampled as contour models C1 and C2. These are finally used as Active Contour Model for segmentation (Figure taken from [39]).

The echogenicity of the SN is a relevant feature in the diagnosis of Parkinson's disease [43]. Computer-guided delineation of SN in TCS images has been the goal of the work presented in [39]. We observed that the butterfly-shaped midbrain section imaged by TCS is fairly invariant across patients as is the SN location relative to the midbrain. Since image artifacts inhibit a direct segmentation of the SN on the basis of low level image features, such as intensity or gradients, we used an elastically deformable model for the detection of the midbrain. This is again a part-based model, but here we used a hierarchy of morphologies in a sequential fit (see Figure 10). The first layer consisted of two SN regions that were attached to a midbrain shape model on the second layer. The shape model is then used to constrain the final segementation (instead of using the shape itself for segmentation such as in [44]).

Since artifacts and noise severely distort the images, it was not possible to compute smooth and reliable potential functions by linear filtering. To set up external image forces, we used a regional classifier over image regions that are large enough to counteract this effect and provide object boundary information. Three different node potentials were defined to account for the non-deterministic image signal:

- Appearance nodes of all parts have a similar potential function: SN nodes reacted on high echodensity, since the SN typically produces more reflections than surrounding midbrain tissue. The internal nodes of the SN models sample a Gaussian low pass filtered version I* = G_*σ *_∗ I of the image *I *(where *σ *= 2.0), and the intensity forces are **f **= κ∇I*, with *κ *> 0. For the echopoor midbrain, we expect low intensities in the interior and let *κ *< 0.

- The boundary FE nodes did not rely on intensity gradients since these were extremely unreliable. Instead, we computed at each iteration of the model fit a balloon-force **f***_b _= κ_b_***n **in the model instances' current contour normal direction that pulls the nodes towards more robustly estimated inter-tissue boundaries. The magnitude and sign *κ_b _*of the balloon force was defined using regional texture information. We dynamically computed an optimal discriminant between "object" and "background" based on statistics over image intensity samples taken from the inside of the model and from the background. The sampling regions were defined in the image in inward and outward normal directions at the boundary nodes of the model instance.

- Young's modulus was 0.9, Poisson ratio was 0.25 and material density was set to 0.9 for all models

As in the previous section, a deformable shape search computed the best fitting shape by simultaneous optimization of multiple two-layer model instances. In this application, however, the search was performed sequentially on the global (midbrain localization) and local context (detection and segmentation of the SN). After finding the best fitting instance of the midbrain model, multiple instances with different parameterizations of the SN models were aligned to it and matched to the data to detect the hyperintense SN regions. This sequential process was necessary because the SN appears as a stripe-like structure on both branches of midbrain, but regularly exhibits the same echotexture as the adjacent brain tissue. That is, the midbrain serves as "guiding structure" for the contained SN, but for a given TCS image it is neither known in advance whether the SN is clearly visible, nor how much an echodense pattern of the SN extends. The fit of SN models should not influence the deformation of the midbrain model instance.

Successful detection means that the true boundary of the SN will be in the vicinity of the boundary estimate by the SN sub-shapes. A final segmentation should make use of this information (e.g., shape-driven level sets [15] or graph cuts [12]). In the last step of our algorithm, the boundaries of the two deformed SN templates were taken as initial placement of Active Contour Models, which are then locally deformed to precisely adapt to the SN boundaries in the TCS image. The template contours were re-sampled to increase the sampling density and flexibility of the contour models. Internal forces and external balloon forces were set up as described in [45] and above (see [39] for details).

**Figure 11:**
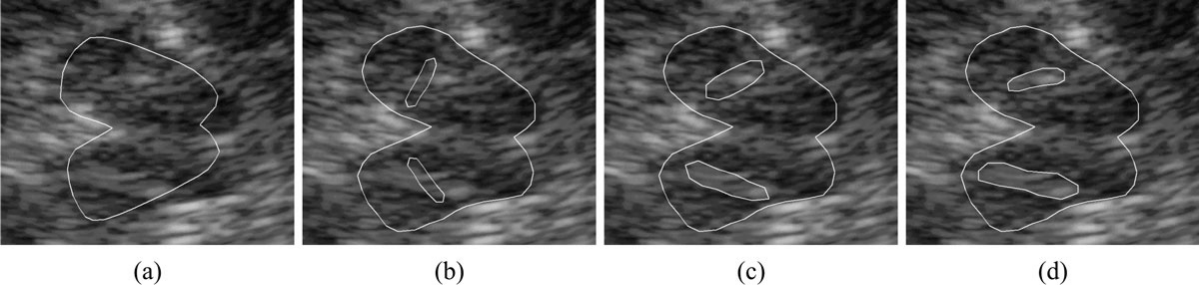
**Example for the fitting process**. (a) Best fitting midbrain model instance (b) induced initialization of the SN model instances (c) deformed SN instances (d) result after active contour segmentation based on (c). (Figure taken from [39]).

The model was applied to 10 data sets, for which expert segmentations were available, and it was found that in all cases the echogenic patterns of the midbrain and SN were correctly localized (Figure 11 shows an example). Small values of 1.03 ± 0.44 mm boundary error at a pixel size of 0.1482 mm showed that the region boundaries were also outlined with high precision. This example application shows how to combine a model search with a constrained segmentation without training based on qualitative a priori knowledge about the appearance of the object. It also shows that the model is able to overcome problems from serious distortions in the image by replacing gradient information by a more elaborate boundary model.

### Detection of human vertebrae in MRI

Investigation of normal variation of the anatomy of the spine and its vertebrae is one of the research questions within SHIP (Study of Health in Pommerania, [46]). We use MRI from SHIP to detect the course of vertebrae of the lower back. More than 40 different MRI image sequences have been acquired within SHIP from several thousands of subjects in Pommerania. Two of the sequences - a T_1_-weighted and a T_2_-weighted sequence - showing spine and vertebrae were used for the detection task using our model-based approach. Although vertebrae detection and segmentation focuses on radiographs and CT images, MRI-based analysis has been the subject of research in medical image analysis as well [47]. The domain knowledge in existing methods is often represented by a specific combination of processing modules where model information is inserted at several stages. Our goal was to investigate whether this can be replaced by our deformable part model. The advantage would be that adapting the detection to some other application would solely relate to model generation without having to change modules of the search process.

Global optimization was not used since initialization is simple for the given data. The user places the model instance in a sagittal view on the middle slice of the image sequence. Optimization then takes place by model deformation based on local image attributes.

**Figure 12:**
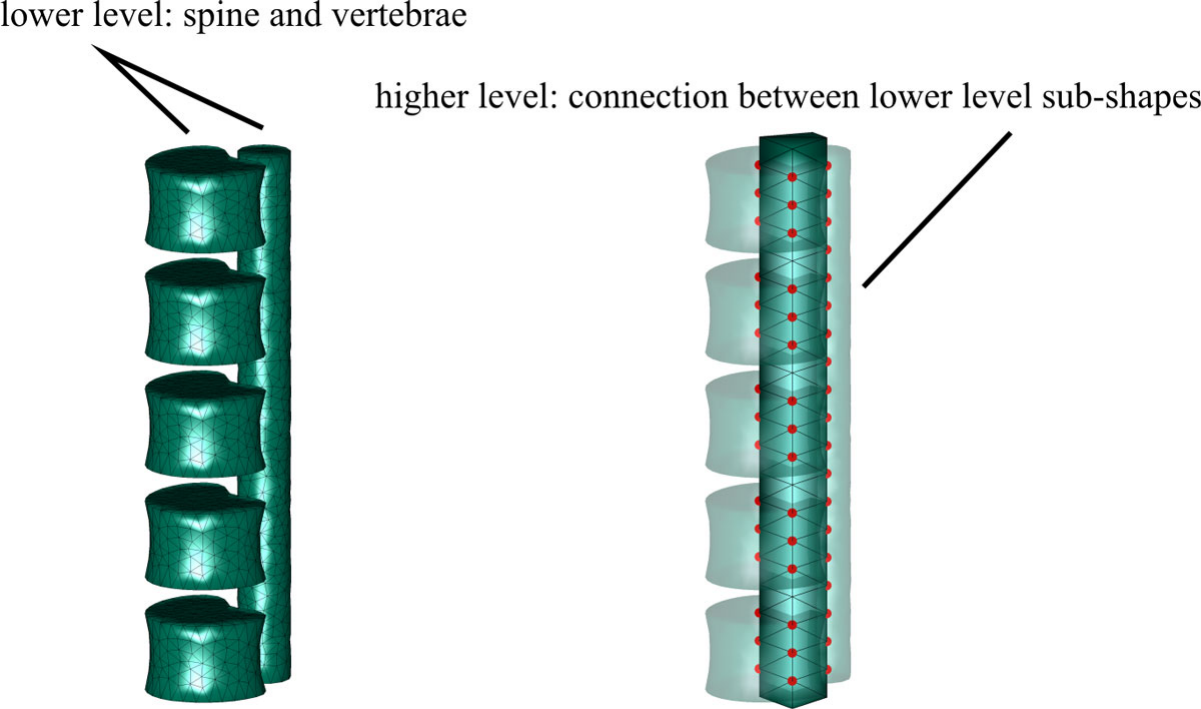
**Two-level FEM for spine and vertebrae**. The morphological sub-shapes are connected at nodes to a structural model that constrains transformation and deformation of sub-shapes with respect to each other.

The model was constructed according to the appearance of vertebrae and spine in a sample image sequence. Main attractor is the spine which is clearly visible in all images. Hence, the model consisted of a two-level hierarchy where vertebrae sub-shapes were connected with a spine sub-shape by a structural model on the second level (see Figure 12). Vertebrae sub-shapes were constructed all equal since no substantial variation is expected between different vertebrae of the lower back. The spine model supported proper localization of the vertebrae. Since its most discriminate aspect was the cylindrical shape, it was represented by a deformable, straight cylinder consisting of appearance nodes only. Appearance nodes represent the vertebra shape, since the relatively low and varying signal of the gradient allows only for small values of *σ *in the smoothing function if boundary nodes were chosen. This strategy bears some similarity to the boundary model that was employed in the previous example for localizing the midbrain in TCS.

For each of the two shape models, the vertebra and the spine, a weighted combination of the T_1_-weighted and the T_2_-weighted image was computed as appearance input (Figure 3 shows computation of the vertebra potential function). Weights for each of the two models were determined a priori and produced a clearly recognizable local minimum for vertebra and spine appearance, respectively.

We used a single heterogeneous model, since the sub-shapes formed a common unit (the spine model) for which, e.g., vibration modes are computed and selected. The restriction that sub-shapes cannot rotate independently around a single connection was not critical. It was even a desired attribute since it very well reflected anatomical relations between sub-shapes. Young's modulus was 1.0, Poisson ratio was 0.0 and material density was set to 0.1 for all models.

Model generation from the sample shape was by Delauney triangulation from evenly distributed sample nodes. It produces a set of "well-shaped" tetrahedrons. Computing the rotation using node warping was solved by minimizing equation 9. Total computation time until convergence on a standard quad-core CPU was between 1.1 and 2.6 seconds per case with an average computation time of 1.5 seconds.

**Figure 13:**
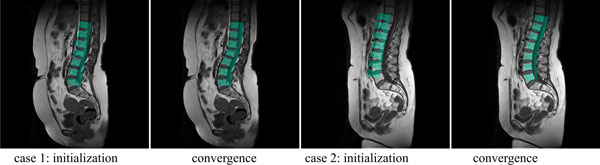
**Examples for detection results (initialization and result after convergence)**. Presently, the model does not contain the typical curvature close to the os sacrum. Detection was satisfactory but orientation of L5 was sometimes not correct when curvature was not supported from a strong signal at the spine (e.g., case 1). Including expected curvature of the spine might be necessary if the detection result is to be used as shape prior to segmentation.

We evaluated the method on 49 data sets from SHIP. Detection was declared successful if the center of each vertebra sub-shape was in the corresponding vertebra in the image data. Examples of results can be seen in Figure 13. Vertebrae were detected correctly in all but one case. In further 3 cases, minor mis-orientations happened. E = 1 provided an almost stationary behavior with respect to detection for parameter changes of more than 10% for the other parameters. Further details on results can be taken from [38].

**Figure 14:**
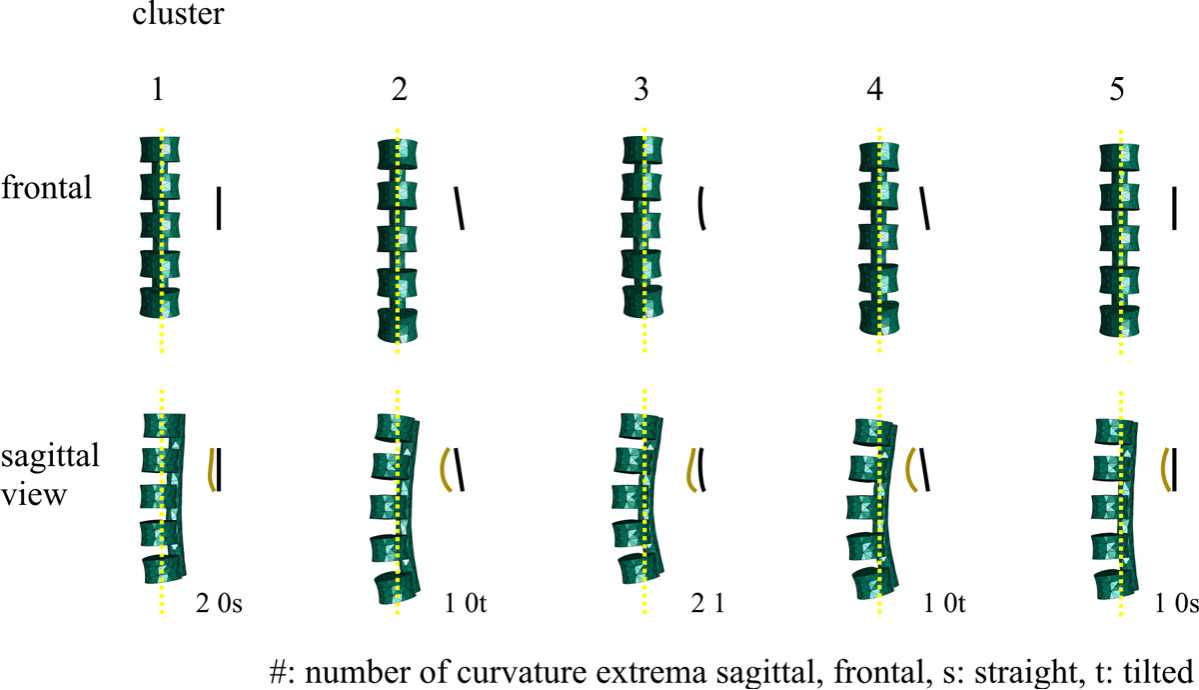
**First results from clustering the converged model**. It shows the ability to distinguish between shape classes. Improvement is needed, however, since insufficient fit in the lower part of the spine degrades the results.

Since one of the analysis goals is to investigate whether variants exist in a normal population, we also clustered shape information of the spinal canal [48]. We currently also investigate clustering on the entire model using the weight vectors of the fitted model instances for analysis of the shape of the spinal cord. This is future work, of course, since detection is not yet followed by segmentation. However, first results are promising, since clustering weight vectors into five clusters using k-means clustering revealed four shape variants that seem to be anatomically meaningful (see Figure 14). Cluster variance, however, is relatively high compared to inter-cluster distances (see Table 1)

**Table 1:** Intra- and inter-cluster distances for shape clustering of the adapted spine model.

Inter-cluster distance	1	2	3	4	5
1	0	73.2748	169.9500	123.3598	81.9592

2	73.2748	0	150.4898	83.5072	88.7331

3	169.9500	150.4898	0	71.1408	90.5465

4	123.3598	83.5072	71.1408	0	66.5185

5	81.9592	88.7331	90.5465	66.5185	0

Intra-cluster distance	63.8863	60.1210	51.8204	50.8078	50.7387

Inter-cluster distances are distances between the means of weights of vibration modes for each cluster. Intra-cluster distances are average standard deviations of all vibration mode weights.

## Conclusion

We presented a part-based deformable model that can be used for directly and efficiently representing domain knowledge necessary for detection of objects in medical images. Different examples demonstrate its application to context-based detection, model-based segmentation, and to shape analysis.

Establishing and applying such model requires readily available methods for generating a triangulated shape and appearance models from a segmentation as well as methods for local and global optimization. Domain knowledge of our part-based deformable model resides almost completely in the model and not in the sequence of processes to fit the model to the data. The model does not require training, since the few parameters (stiffness, decomposition into parts, node potentials, pose parameters) can be set a priori and adapted intuitively. However, experience about setting up the model will speed up the design process. A number of decisions have to be made:

- Structural decomposition of the model depends on part-relationships that can be derived from knowledge about the anatomy or arrangement objects to be detected. In medical applications, parts are typically anatomic substructures or a group of neighboring landmark structures that are necessary to determine an object's position.

- Elasticity parameters describe the variability between shape instances and - on the structural level - variability of spatial relations between sub-shapes.

- External forces are set depending on the expected appearance of the object in the image. While gradient-based potential forces often allow for exact determination of the object boundary, they may be supplemented by intensity or texture-based forces if gradient information is unreliable.

In future we will investigate the potential for parameter training of the model. Training of the few parameters in the data and the model terms should be much simpler requiring less training data than training of a PDM. An alternative to adapt parameters would be to take the detection result as an initialization for the search of an optimal potential field given the converged model instance instead of trying to improve the overall performance of the model. This may also enhance the potential of the model to guide data-driven segmentation schemes such as level set segmentation or graph cuts. It will also be worthwhile to take a second look at the analysis of shape parameters for the detection of shape classes using adapted model parameters.

## Competing interests

The authors declare that they have no competing interests.

## Authors' contributions

KDT supervised the work of MR and KE and conceived of the paper, MR developed and investigated the 3-D integrated FEM, KE developed and investigated the hierarchical FEM, all authors contributed to the draft of the manuscript, all authors read and approved the final manuscript.

## Declarations

Funding for this article came from the DFG priority program 1335: Scalable Visual Analytics (grant TO 166/13-2).

This article has been published as part of *BioMedical Engineering OnLine *Volume 13 Supplement 1, 2014: Selected articles from the 35th Annual International Conference of the IEEE Engineering in Medicine and Biology Society: Workshop on Current Challenging Image Analysis and Information Processing in Life Sciences. The full contents of the supplement are available online at http://www.biomedical-engineering-online.com/supplements/13/S1

## References

[B1] Hill A Taylor CJ Model-based image interpretation using genetic algorithms Image and Vision Computing 1992 10 5 295 300 10.1016/0262-8856(92)90045-5

[B2] Gloger O Toennies KD Liebscher V Kugelmann B Laqua R Völzke H Prior shape level set segmentation on multistep generated probability maps of MR datasets for fully automatic kidney parenchyma volumetry IEEE Trans Medical Imaging 2012 31 2 312 325 10.1109/TMI.2011.2168609 21937343

[B3] Stegmann MB Ersbøll BK Larsen R FAME--a flexible appearance modeling environment IEEE Trans Medical Imaging 2003 22 10 1319 1330 10.1109/TMI.2003.817780 14552585

[B4] Hojjatoleslami SA Kittler J Region growing: a new approach IEEE Trans Image Processing 1998 7 7 1079 1084 10.1109/83.701170 18276325

[B5] McInerney T Terzopoulos D Deformable models in medical image analysis: a survey Medical Image Analysis 1996 1 2 91 108 10.1016/S1361-8415(96)80007-7 9873923

[B6] Hamarneh G Yang J McIntosh C Langille M Fitzpatrick JM, Reinhardt JM 3D live-wire-based semi-automatic segmentation of medical images Proc. SPIE 5747, Medical Imaging 2005: 12-17 February 2005; San Diego 2005 International Society for Optics and Photonics 1597 1603

[B7] Manniesing R Viergever MA Niessen WJ Vessel enhancing diffusion: A scale space representation of vessel structures Medical Image Analysis 2006 10 6 815 825 10.1016/j.media.2006.06.003 16876462

[B8] Tao Y Lu L Dewan M Chen AY Corso J Xuan J Salganicoff M Yang GZ, Hawkes D, Rueckert D, Noble A, Taylor C Multi-level ground glass nodule detection and segmentation in CT lung images Medical Image Computing and Computer-Assisted Intervention - MICCAI 2009: 20-24 September 2009; London; LNCS 2009 5762 London: Springer 715 723 10.1007/978-3-642-04271-3_87 20426175

[B9] Lalonde M Beaulieu M Gagnon L Fast and robust optic disc detection using pyramidal decomposition and Hausdorff-based template matching IEEE Trans Medical Imaging 2001 20 11 1193 1200 10.1109/42.963823 11700746

[B10] Golemati S Stoitsis J Sifakis EG Balkizas T Nikita KS Using the Hough transform to segment ultrasound images of longitudinal and transverse sections of the carotid artery Ultrasound in Medicine & Biology 2007 33 12 1918 1932 10.1016/j.ultrasmedbio.2007.05.021 17651891

[B11] Friedman D Zhang T Schmid C, Soatto S, Tomasi C Interactive graph cut based segmentation with shape priors IEEE Conf Computer Vision and Pattern Recognition CVPR 2005: 20-25 June 2005; San Diego 2005 1 Los Alamitos: IEEE Computer Society Press 755 762

[B12] El-Zehiry N Elmaghraby A Graph cut based deformable model with statistical shape priors19th IEEE Intl Conf Pattern Recognition ICPR 2008: 8.-11. December 2008 2008 Tampa. IEEE 1146 1149

[B13] Veksler O Forsyth D, Torr P, Zisserman A Star shape prior for graph-cut image segmentation European Conference on Computer Vision ECCV 2008: 12-18 October 2008; Marseille; LNCS 2008 2351 London: Springer 78 92

[B14] Rousson M Paragios N Heyden A, Sparr G, Nielsen M, Johansen P Shape priors for level set representations European Conference on Computer Vision ECCV 2002: 7 May - 2 June 2002; Copenhagen. LNCS 2002 2351 London: Springer 78 92

[B15] Cremers D Osher SJ Soatto S Kernel density estimation and intrinsic alignment for shape priors in level set segmentation Intl J Computer Vision 2006 69 3 335 351 10.1007/s11263-006-7533-5

[B16] Cootes TF Taylor CJ Hogg D, Boyle R Active shape models -- 'smart snakes' British Machine Vision Conference BMVC92: 22-24 September 1992 1992 London: Springer 266 275

[B17] Cootes TF Taylor CJ Cooper DH Graham J Active shape models - their training and application Computer Vision and Image Understanding 1995 61 1 38 59 10.1006/cviu.1995.1004

[B18] Cootes TF Hill A Taylor CJ Haslam J Use of active shape models for locating structures in medical images Image and Vision Computing 1994 12 6 355 365 10.1016/0262-8856(94)90060-4

[B19] van Ginneken B Frangi AF Staal JJ ter Haar Romeny BM Viergever MA Active shape model segmentation with optimal features IEEE Trans Medical Imaging 2002 21 8 924 933 10.1109/TMI.2002.803121 12472265

[B20] Ruppertshofen H Lorenz C Schmidt S Beyerlein P Salah Z Rose G Schramm H Discriminative Generalized Hough transform for localization of joints in the lower extremities Computer Science - Research and Development 2011 26 1-2 97 105 10.1007/s00450-010-0137-x

[B21] Cohen LD Cohen I Finite-element methods for active contour models and balloons for 2-D and 3-D images IEEE Trans Pattern Recognition and Machine Intelligence 1993 15 11 1131 1147 10.1109/34.244675

[B22] Dornheim L Toennies KD Dixon K Duncan J, Gerig G Automatic segmentation of the left ventricle in 3d SPECT data by registration with a dynamic anatomic model Medical Image Computing and Computer-Assisted Intervention - MICCAI 2005: 26-29 October 2005; Palm Springs; LNCS 2005 3749 London: Springer 335 342 16685863 10.1007/11566465_42

[B23] Shen T Li H Huang X Active volume models for medical image segmentation IEEE Trans Medical Imaging 2011 30 3 774 791 10.1109/TMI.2010.2094623 21118771

[B24] Felzenszwalb PF Huttenlocher DP Pictorial structures for object recognition Intl J Computer Vision 2005 61 1 55 79

[B25] Wu B Nevatia R Detection of multiple, partially occluded humans in a single image by Bayesian combination of edgelet part detectors Intl Conf Computer Vision - ICCV 2005: 17-21 October 2005 2005 1 Beijing. IEEE Computer Society 90 97

[B26] Crandall DJ Huttenlocher DP Leonardis A, Bischof H, Pinz A Weakly supervised learning of part-based spatial models for visual object recognition Eur. Conf. Computer Vision ECCV 2006: 7-13 May 2006; Graz; LNCS 2006 3951 Berlin Heidelberg: Springer 16 29

[B27] Bajcsy R Lieberson R Reivich M A computerized system for the elastic matching of deformed radiographic images to idealized atlas images J Comp Ass Tomography 1983 7 4 618 625 10.1097/00004728-198308000-00008 6602820

[B28] Petyt M Introduction to finite element vibration analysis 1998 Cambridge University Press

[B29] Oh SI Altan T Metal forming and the finite-element method 1989 Oxford University Press

[B30] Sclaroff S Deformable prototypes for encoding shape categories in image databases Pattern Recognition 1997 30 4 627 641 10.1016/S0031-3203(96)00108-2

[B31] Sclaroff S Pentland AP Modal matching for correspondence and recognition IEEE Trans Pattern Analysis and Machine Intelligence 1995 17 6 545 561 10.1109/34.387502

[B32] Cootes TF Taylor CJ Combining point distribution models with shape models based on finite element analysis Image and Vision Computing 1995 13 5 403 409 10.1016/0262-8856(95)99727-I

[B33] Choi MG Hyeong-Seok K Modal warping: Real-time simulation of large rotational deformation and manipulation IEEE Trans Visualization and Computer Graphics 2005 11 1 91 101 10.1109/TVCG.2005.13 15631132

[B34] Müller M Gross M Interactive virtual materials Proc Graphics Interface GI'04 2004 239 246

[B35] Byers R Xu H A new scaling for Newton's iteration for the polar decomposition and its backward stability SIAM J Matrix Analysis and Applications 2008 30 2 822 843 10.1137/070699895

[B36] Engel K Brechmann A Toennies KD A two-level dynamic model for the representation and recognition of cortical folding patterns19th IEEE Intl Conf Image Processing ICIP2005: 11-14 September 2005 2005 2 Genoa. IEEE Press 297 300

[B37] Engel K Toennies KD Hierarchical vibrations for part-based recognition of complex objects Pattern Recognition 2010 43 8 2681 2691 10.1016/j.patcog.2010.02.009

[B38] Rak M Engel K Toennies KD Bronstein M, Favre J, Hormann K Closed-form hierarchical finite element models for part-based object detection Vision, Modeling, and Visualization 2013: 11-13 September 2013; Lugano 2013 Eurographics Association

[B39] Engel K Toennies KD Segmentation of the midbrain in transcranial sonographies using a two-component deformable model Annals of the BMVA 2009 2009 4 1 13

[B40] Engel K Toennies KD Brechmann A Part-based localisation and segmentation of landmark-related auditory cortical regions Pattern Recognition 2011 44 2017 2033 10.1016/j.patcog.2010.09.004

[B41] Fischl B Sereno MI Dale AM Cortical surface-based analysis: II: Inflation, flattening, and a surface-based coordinate system Neuroimage 1999 9 2 195 207 10.1006/nimg.1998.0396 9931269

[B42] Tailarach J Tournoux P A Coplanar Stereotaxic Atlas of the Human Brain: An Approach to Medical Cerebral Imaging 1988 Thieme, New York

[B43] Berg D Jabs B Merschdorf U Beckmann H Becker G Echogenicity of substantia nigra determined by transcranial ultrasound correlates with severity of parkinsonian symptoms induced by neuroleptic therapy Biol Psychiatry 2001 50 6 463 467 10.1016/S0006-3223(01)01190-8 11566164

[B44] Hishida H Suzuki H Michikawa T Ohtake Y Oota S CT image segmentation using FEM with optimized boundary condition PLoS One 2012 7 2 e31116 10.1371/journal.pone.0031116 22389668 PMC3289631

[B45] Lobregt S Viergever MA A discrete dynamic contour model IEEE Trans Medical Imaging 1995 14 1 12 24 10.1109/42.370398 18215806

[B46] Völzke H Alte D Schmidt CO Radke D Lorbeer R Friedrich N Aumann N Lau K Piontek M Born G Havemann C Ittermann T Schipf S Haring R Baumeister SE Wallaschofski H Nauck M Frick S Arnold A Jünger M Mayerle J Kraft M Lerch MM Dörr M Reffelmann T Empen K Felix SB Obst A Koch B Gläser S Cohort profile: The study of health in Pomerania Intl J Epidemiology 2011 40 2 294 307 10.1093/ije/dyp394 20167617

[B47] Huang SH Chu YH Lai SH Novak CL Learning-based vertebra detection and iterative normalized-cut segmentation for spinal MRI IEEE Trans Medical Imaging 2009 28 10 1595 1605 10.1109/TMI.2009.2023362 19783497

[B48] Klemm P Lawonn K Rak M Preim B Tönnies KD Hegenscheid K Völzke H Oeltze S Bronstein M, Favre J, Hormann K Visualization and analysis of lumbar spine canal variability in cohort study data Vision, Modeling, and Visualization 2013: 11-13 September 2013; Lugano 2013 Eurographics Association

